# High-performance position-sensitive detector based on the lateral photoelectrical effect of two-dimensional materials

**DOI:** 10.1038/s41377-020-0307-y

**Published:** 2020-05-20

**Authors:** Chang Hu, Xianjie Wang, Bo Song

**Affiliations:** 1 Department of Physics, Harbin Institute of Technology, 150001 Harbin, China; 2 National Key Laboratory of Science and Technology on Advanced Composites in Special Environments, Harbin Institute of Technology, 150001 Harbin, China

**Keywords:** Optics and photonics, Optical sensors

## Abstract

Two-dimensional (2D) materials such as graphene and transition-metal chalcogenides have been extensively studied because of their superior electronic and optical properties. Recently, 2D materials have shown great practical application in position-sensitive detectors (PSDs), originating from the lateral photoelectrical effect of the materials or junctions. The high position sensitivity and ultrafast photoresponse of PSDs based on 2D materials, especially compatibility with Si technology, may enable diverse optoelectronic applications. In this review, recent studies of PSDs based on 2D materials are summarized, providing a promising route for high-performance PSDs.

## 2D materials: position-sensitive detectors

Photodetectors that can monitor the location of an incident laser spot are useful for applications such as laser beam alignment and stabilization. While silicon p-n or p-i-n detectors are commonly used, Chang Hu, Xianjie Wang and Bo Song from Harbin Institute of Technology in China, have now reviewed the advantages and capabilities of using a variety of 2D materials for performing this task. They report how junctions based on pairing graphene or molybdenum disulfide with silicon can result in positive-sensitive detectors can be highly sensitive and offer an ultrafast response. The good performance is thought to be due to the high charge mobility and strong absorption of the 2D materials. Future challenges include extending the wavelength range of operation of such detectors beyond the visible to the near-infrared and ultraviolet.

## Introduction

**Fig. 1:**
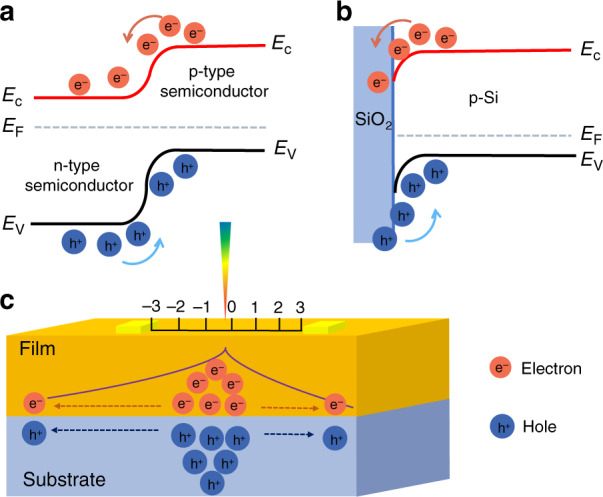
Schematic diagram of PSD principle. **a** Schematic diagram of the energy band structure of PSD based on the *p*–*n* junction and **b** energy band structure of PSD based on the SiO_2_/Si substrate. **c** Schematic diagram of the carrier lateral diffusion model

Since Schottky and Wallmark discovered and promoted the lateral photovoltaic effect (LPE) in *p*–*n* junctions^1^, intensive research has been conducted on the LPE in diverse applications, including space exploration, environmental monitoring, and optical engineering. In the LPE, when one side of a *p*–*n* junction is illuminated by a light spot, the optically excited electron-hole pairs will be separated up and down by the built-in electric field. Notably, the formation mechanism of the built-in field in position-sensitive detectors (PSDs) with different architectures is different. As shown in Fig. 1a, the PSD based on a *p*–*n* junction uses the built-in field formed at the interface due to different band structures to separate the photogenerated carriers. The built-in field in the PSD based on the insulator-coated substrate (such as a graphene transistor on a SiO_2_/Si substrate) is formed by band energy bending induced by the localized interface states, and the photogenerated carriers accumulate at the SiO_2_/Si interface and then diffuse laterally, as shown in Fig. 1b. The mechanism of carrier lateral diffusion can be explained by the model shown in Fig. 1c. The photogenerated electrons enter the *n*-type semiconductor, while the photogenerated holes enter the *p*-type semiconductor. Then, these generated carriers diffuse laterally from the light spot to balance the electric field along the layer^2–6^. In general, the relationship between the total number of photogenerated electrons *n*_0_ and that of photogenerated electrons transmitted into *n*-type semiconductor *N*_0_ can be described as follows:

1 $$N_{\mathrm{0}} = n_{\mathrm{0}}\left[ {1 - P^{\tau p/n_{\mathrm{0}}}} \right]$$

where *P* is the probability of photogenerated electrons entering the *n*-type semiconductor, *p* is the laser power, and *τ* is a time-dependent coefficient.

The electron diffusion equation at position *r* is

2 $$D_{\mathrm{n}}\frac{{d^{\mathrm{2}}N(r)}}{{{\mathrm{dr}}^{\mathrm{2}}}} = \frac{{N(r)}}{{{\uptau }}_{\mathrm{n}}}$$

From these equations, the electron density *N*(r) can be derived as follows:

3 $$N\left( {\mathrm{r}} \right) = N_{\mathrm{0}}{\mathrm{exp}}\left( {{\mathrm{ - }}\frac{{|x - r|}}{{{\uplambda }}_{\mathrm{n}}}} \right)$$

where ${\uplambda}_{\mathrm{n}} = \sqrt {D_{\mathrm{n}}{\uptau}_{\mathrm{n}}}$ is the diffusion length, $D_{\mathrm{n}} = \frac{{k_{\mathrm{B}}T}}{{{\mathrm{e}}^{\mathrm{2}}\rho N_{{\mathrm{F}}0}}},$ and *ρ* are the diffusion constant and resistivity of the *n*-type semiconductor, respectively; *τ*_n_ is the electron diffusion lifetime, $N_{{\mathrm{F}}0} = \frac{{8\pi }}{3}\left( {\frac{{2m_{\mathrm{e}}E_{{\mathrm{F}}0}}}{{h^2}}} \right)^{\frac{3}{2}}$ is the electron density below the Fermi level (*E*_F0_), and *x* is the position of the laser point.

The Fermi level of an *n*-type semiconductor after laser irradiation at position r can be expressed as follows:

4 $$E_{{\mathrm{Fn}}}\left( r \right) = E_{{\mathrm{F}}0} + \frac{{k_{\mathrm{B}}T}}{{n_{\mathrm{T}}}}N(r)$$

where the electron density in the n-type semiconductor conduction band is

$$n_{\mathrm{T}} = \frac{{{\mathrm{2}}\left( {2\pi m_{\mathrm{e}}k_{\mathrm{B}}T} \right)^{\frac{3}{2}}}}{{h^3}}{\mathrm{exp}}\left( {{\mathrm{ - }}\frac{{E_{\mathrm{C}}- E_{{\mathrm{F}}0}}}{{k_{\mathrm{B}}T}}} \right)$$

Using the above formulas, a theoretical formula for the relationship between the LPE and laser irradiation position can be obtained as follows:

5 $${\mathrm{LPE}} = \frac{{E_{{\mathrm{Fn}}}\left( {\mathrm{L}} \right) - E_{{\mathrm{Fn}}}\left( { - L} \right)}}{e} = {\mathrm{Kn}}_0\left[ {{\mathrm{exp}}\left( {{\mathrm{ - }}\frac{{|L - x|}}{{\lambda _{\mathrm{n}}}}} \right) - {\mathrm{exp}}\left( {{\mathrm{ - }}\frac{{|L + x|}}{{\lambda _{\mathrm{n}}}}} \right)} \right]$$

where coefficient $K = \frac{{k_{\mathrm{B}}T}}{{{\mathrm{en}}_{\mathrm{T}}}},$ and *L* and –*L* are the positions of the two electrodes. Therefore, the voltage difference detected in the two terminal electrodes has a good linear relationship with the laser spot position, making it suitable for PSDs that can detect very small displacements. Silicon-based *p*–*n* or *p*-i-*n* junctions are the most commonly used structures for current PSDs, and the response speed was increased from milliseconds in amorphous silicon to microseconds in crystalline silicon devices^7^. PSDs with various architectures have been reported in recent decades, including metal-semiconductor junctions^8–13^, hydrogenated amorphous silicon structures^14^, perovskite materials^15–21^, and two-dimensional (2D) materials^22–24^.

The energy band structure and carrier mobility of materials are important factors for the lateral diffusion mechanism of photogenerated carriers. A 2D material is defined as atomically thin layered crystalline sheets with van der Waals interactions between the layers. Graphene and several groups of transition-metal chalcogenides are attractive materials because of their high carrier mobility, tunable energy bandgap, and large light absorptivity^25–30^; these materials have been prepared and successfully developed as field-effect transistors^31,32^, photocatalysts^33,34^, gas sensors^35^, and photodetectors^36–38^ over the past decade. Until now, numerous studies have shown that 2D materials have great application potential in PSDs^25,26,39–42^. The PSDs based on 2D materials exhibit outstanding properties in a broadband wavelength range with a position sensitivity of up to 401 mV mm^−1^ ^43^, ultrafast response time of less than 0.5 μs^43^, nonlinearity of no more than ~2%^44^, and a power detection limit as low as ~17 nW^6^. Table 1 shows the performance parameters of various PSDs; obviously, the 2D material-based PSDs satisfy the urgent requirement for highly sensitive, ultrafast photo detection, and low laser power.

**Table 1:** Performance parameters of Si-based PSDs

Structure	Laser wavelength (nm)	Laser power (mW)	Position sensitivity (mV mm^−1^ mW^−1^)	Response time (μs)
Cr/SiO_2_/Si^12^	635	5	8.40	\
Co/Si^13^	832	5	16.40	\
Ti/TiO_2_/Si^10^	632	3	37.67	\
a-Si:H/c-Si^14^	980	8.3	0.88	\
Graphene/SiO_2_/Si^25^	514	5 × 10^−5^	\	1.2
Graphene/Si^6^	532	82 × 10^−5^	365.85	0.44
a-MoS_2_/Si^26^	780	10	18.3	2.1

Herein, a systematic overview of recent studies on 2D material-based PSDs is provided. The applications of graphene and transition-metal sulfides in PSDs are introduced. The physical mechanism of the large position sensitivity and ultrafast response is elucidated, facilitating new PSD designs.

## Graphene-based PSDs

**Fig. 2:**
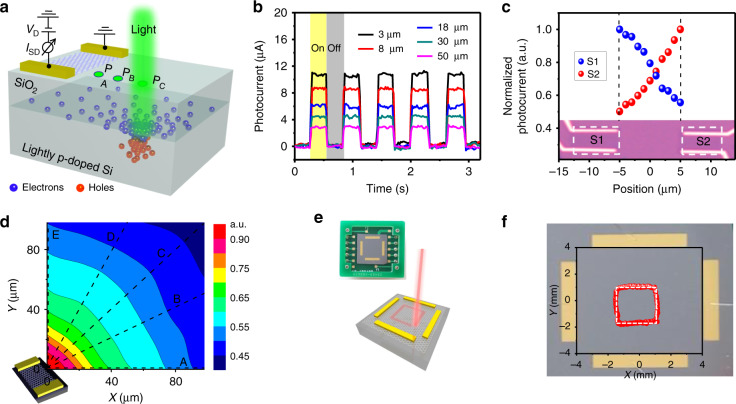
Graphene-based PSD. **a** Schematic diagram of graphene-based PSD. **b** Photoswitching characteristics of graphene-based PSD at different light illumination positions. **c** Position dependence of the photoresponse of a one-dimensional PSD prepared under 50nW incident light. **d** 2D spatial mapping of the photocurrent of PSD under 400nW incident light. **e** Optical image of a 2D PSD and schematic diagram of the movement of light in the operating area. **f** Measured trajectory (red dots) of the laser. The white dotted line represents the actual position^6,25^

Graphene consists of a single layer of sp^2^-hybridized carbon atoms with excellent mechanical, thermal, electrical properties, high chemical stability, and high carrier mobility. Graphene-based devices typically exhibit excellent performance, such as a large photoresponsivity, a high detectivity, a great power conversion efficiency, and an ultrafast response speed. Wang et al. reported a graphene-based PSD that can detect ultrafast weak signals because of the high mobility of graphene and long lifetime of photogenerated carriers at the interface of SiO_2_/Si^25^. Figure 2a shows a lightly p-doped Si substrate with a SiO_2_ layer, and two-terminal graphene is deposited on the top. When light is incident on the surface of the detector, the photogenerated electron-hole pairs are separated by the built-in electric field at the interface of SiO_2_ and Si and then spread laterally in the depletion layer. The positional information can be obtained by the correspondence between photoelectric signals collected by the electrodes on graphene and the position of light irradiation. The electrons occupying the acceptor level decrease and the holes occupying the donor level increase due to a large positively charged state from the lightly *p*-doped Si band gap existing at the oxide-silicon interface, making the energy band at the interface bend down. The electrons in the Si conduction band gather at the interface with a lower potential to form a negative depletion layer near the silicon surface; thus, an intrinsic built-in field that can separate the photogenerated carriers is formed.

Thus, the electrons will migrate to the SiO_2_/Si interface and then diffuse laterally until equilibrium is reached, and the magnitude of the photocurrent decreases exponentially with increasing distance from the laser spot. Electrons diffusing into the underlying region of graphene change the hole concentration and channel current by capacitive coupling. This gate effect results in an ultrahigh interfacial amplification (*G*), determined by the ratio of the lifetime (*τ*_l_) of the photogenerated carrier at the SiO_2_/Si interface and the transit time (*τ*_l_) in the graphene channel between the two electrodes, and *G* = *τ*_l_/*τ*_t_. Ultrahigh mobility graphene (approximately 8000 cm^2^ V^−1^ S^−1^) and the long carrier lifetime of SiO_2_/Si (10^−6^ s) in the graphene-based PSD result in an extremely high G (approximately 10^4^), thus significantly improving the sensitivity of the device. This interfacial amplification process behaves similarly to a built-in amplifier, which does not increase the signal noise in the graphene channel, so it can significantly improve the sensitivity of the device.

Figure 2b shows the photoswitching characteristics of graphene-based PSD at different light illumination positions and the photocurrent signals collected by the electrodes on the graphene. When the laser spot is far away from the graphene, the photocurrent is close to zero because of the isotropic diffusion of carriers, whereas the photocurrent increases when the laser position is close to the graphene channel.

Figure 2c shows the photoresponse characteristics of the PSD under 50 nW laser irradiation with a wavelength of 514 nm. The inset shows the optical image of two devices (S1 and S2) on the same substrate separated by 10 μm. For device S1, as the laser position increases from −5 to 5 μm from the graphene channel, the normalized photocurrent (*I*_2_ − *I*_1_)/(*I*_2_ + *I*_1_) decreases from ∼1 to 0.5, while S2 increases from 0.5 to 1. Obviously, the photocurrent of graphene-based PSD performs well. Furthermore, it can also detect the positional changes in 2D space. Figure 2d shows the distribution of photocurrent at different locations. Because the detection limit of photocurrent is ∼0.5 μA, the spatial position resolution of PSD should be less than 1 μm.

Figure 2e, f show optical images of another graphene-based 2D PSD with a different structure^6^. In this work, Wang et al. designed a high-performance passive PSD based on a graphene-Si hybrid structure. These authors transferred a large area of monolayer graphene to the surface of a lightly *n*-doped Si substrate and produced a prototype PSD with a size of 8 × 8 mm. Different from the PSD in Fig. 2a, the pinning effect will bend the energy band of the *n*-doped Si surface upward so that the photogenerated holes will enter the graphene layer under the action of the built-in field. A 635-nm laser was used to illuminate the device along a square track (red dots), and the signals collected by the two pairs of electrodes can determine the position of the laser spot in real time (white dots). The red and white dashed squares match; subsequent calculation processing and converting the electrode into a pillow shape can effectively reduce the measurement error. Moreover, the presence of a built-in field in this PSD can effectively separate photogenerated carriers without the need for an external voltage, i.e., there is no extra power consumption during the measurement, making it suitable for portable and integrated devices.

**Fig. 3:**
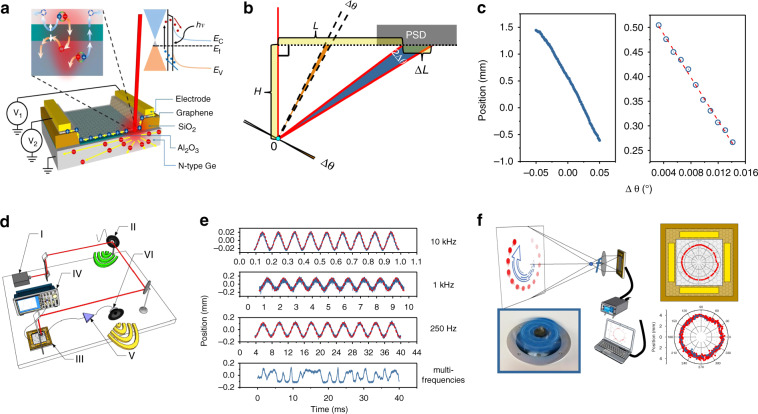
Multiple applications of graphene-based PSDs. **a** Schematic diagram of the graphene-Ge PSD. **b** Schematic structure of triangulation angle measurement by the PSD. **c** Results of the small-angle measurement. **d** Setup of the vibration frequency measurement. **e** Different vibrations recorded by the PSD. **f** High-speed trajectory tracking using the graphene-Ge PSD^45^

Liu et al. further showed a large-area graphene-Ge Schottky heterojunction^45^. Large-area monolayer graphene (5 × 5 mm^2^) is deposited on the *n*-type Ge substrate, as shown in Fig. 3a. The pinning effect originating from the surface state causes the energy band of the depletion layer to bend upward, forming a built-in field with the direction from the Ge substrate to the surface and providing a greater signal collection efficiency for the PSDs. Graphene itself can also absorb light and produce photogenerated carriers, extending the operating wavelength to the near-infrared region (up to 1600 nm). The highest position sensitivity of this PSD under 1550 nm laser irradiation is approximately 50 mV/mm, and the voltage noise is approximately 4 μV, which is almost negligible. In addition to detecting positional changes, PSDs have many novel features in various applications^45^. As shown in Fig. 3b, angular changes can also be accurately measured with the clever modification of the PSD. When a laser beam is illuminated on the object, the reflected light can be detected by the PSD. If the object rotates at a small angle Δ*θ*, then the corresponding reflected light will move Δ*L* on the PSD, satisfying the relationship: $\Delta {\uptheta} = \frac{1}{2}\left[ {{\mathrm{arctan}}\left( {\frac{{L + \Delta L}}{H}} \right) - {\mathrm{arctan}}\left( {\frac{L}{H}} \right)} \right]$. By measuring the displacement Δ*L* on the PSD, the angular difference can be obtained. Figure 3c shows that Δ*θ* is linearly related to Δ*L* in the range from −0.05 to 0.05 degrees. According to the high spatial resolution (0.1 μm) of graphene-based PSD, the smallest detectable angular difference can be accurate to 5 × 10^−6^ degrees within a working distance of 50 cm.

The fast response characteristics of the PSD can also be used to precisely distinguish the frequency of sound, as shown in Fig. 3d. In this noncontact optical sensing system, the PSD can precisely measure and recover the sound frequency by the following process: The laser beam (I) is irradiated on the speaker surface (II), and the vibration of the speaker causes a small displacement of the reflected light. This displacement can be detected by the PSD (III) and recorded by an oscilloscope (IV). The recorded electrical signal can be converted into sound again through an amplifier (V) and speaker (VI). Figure 3e shows the experimental results of the PSD-based sound recording and storage system. The trajectory tracking of high-speed moving objects has detector requirements of high accuracy and response speed. Figure 3f shows that the graphene-based PSD can track the trajectory of a target moving at a speed higher than 100 km/h. The positions of the object (blue dots) can be clearly recorded. Without the limitations of the source meter and software system, the minimum capture interval can be less than 110 μs. This trajectory tracking has a high spatial resolution and precise trajectory reproduction because the mechanical dither (red dots) can be recorded in real time.

Therefore, a graphene-based PSD has excellent characteristics, such as a fast response time, high sensitivity, good linearity, and no power consumption, making it a promising candidate for various applications in precise measurements and noncontact optical sensing.

## MoS_2_-based PSDs

As a typical transition-metal dichalcogenide, MoS_2_ has become one of the most studied 2D materials in recent years. MoS_2_ has a suitable band gap and a high carrier and light absorbance for optoelectronic devices; however, there are still many problems in preparing large-area and defect-free MoS_2_, making it difficult to fully exert its brilliant optoelectronic properties in devices. To overcome these drawbacks, the preparation of multilayer MoS_2_ has been attempted, and a feasible method has been developed for constructing high-performance optoelectronic devices by changing the morphology of MoS_2_ or by combining it with different semiconductors, such as making MoS_2_/Si and MoS_2_/GaAs junctions^23,40^.

**Fig. 4:**
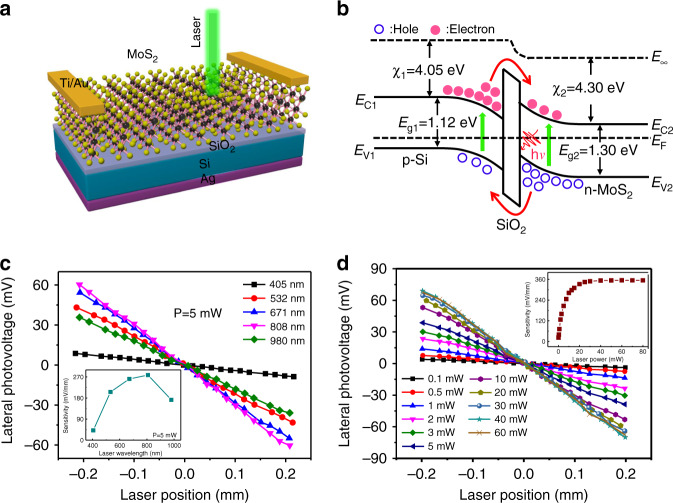
MoS_2_-based PSD. **a** Schematic illustration of the lateral photoelectric effect in the MoS_2_/Si heterojunction. **b** Energy band diagram of the MoS_2_/Si heterojunction. **c** Dependence of the LPE on the laser position for different lasers with an illumination of 5 mW. **d** Dependence of the LPE on the laser position with an illumination of a 532 nm laser at different laser powers^43,44^

Qiao et al. prepared vertically oriented few-layered MoS_2_ nanosheets (V-MoS_2_) using chemical vapor deposition (CVD)^43,44^. These researchers successfully transferred MoS_2_ nanosheets to a Si substrate to form a self-powered, high-performance PSD. Figure 4a shows a schematic illustration of the PSD based on a V-MoS_2_/Si heterojunction. Ti/Au electrodes were deposited on the surface of MoS_2_ nanoplates, and Ag electrodes were plated on the back of the substrate to ensure good signal collection. The band gaps of the p–Si substrate (*E*_g1_) and n-MoS_2_ (*E*_g2_) are 1.12 eV and 1.30 eV, respectively, as shown in Fig. 4b. When a laser beam is incident on the surface of the heterojunction, the film and substrate absorb the energy of the laser to excite electron-hole pairs. Under the action of the built-in field at the interface, the electrons are swept and tunneled to the MoS_2_ layer, and the corresponding holes enter the Si substrate.

Figure 4c shows the typical laser position dependence of the LPE curves of the MoS_2_-based PSD. When the laser beam moved from one electrode to the other, a linear relationship was observed between the laser position and LPE. By adjusting the laser power of different wavelengths to 5 mW during the measurement, it was observed that all the curves maintain good linearity. However, the position sensitivity will first increase with increasing wavelength from 405 to 808 nm and then decrease at a longer wavelength laser irradiation, as shown in the inset of Fig. 4c, indicating that the relationship between the LPE and laser wavelength is nonmonotonic. In addition, the laser power also significantly affects the LPE. Figure 4d shows the LPE curves with different laser powers; the inset shows the relationship between laser power and position sensitivity. When the laser power was increased from 0.1 to 80 mW, the position sensitivity first increases exponentially and then becomes saturated. The MoS_2_ layer has a large bandgap, and its thickness is negligible relative to the Si wafer. Most of the light absorption occurs in the Si wafer; therefore, the PSD has the largest response to an 808 nm wavelength laser. Moreover, the built-in field of heterojunctions has a limited ability to separate the photogenerated carriers. Although an increase in laser power can excite more carriers, it also increases the recombination rate. The number of electron-hole pairs that can be effectively separated is limited. Excess photogenerated carriers recombine quickly, and the LPE cannot be further increased.

Most of the studies believe that high-quality 2D materials are the basis of good LPE performance, but the preparation conditions of a single-crystal MoS_2_ film are very demanding, significantly limiting its application prospects. Compared with a MoS_2_ single-crystal film, amorphous MoS_2_ (a-MoS_2_) can be easily prepared over a large area on a Si wafer. Hu et al. reported a PSD based on a-MoS_2_/Si structures with a large LPE and an ultrafast response time^26^. The a-MoS_2_ films were grown on *p*-type and *n*-type Si wafers to form *p*–*n* and *n*–*n* junctions by pulsed laser deposition, respectively, thus achieving two different junctions with opposite-direction built-in fields at the interfaces. The laser excited holes and electrons flow to the a-MoS_2_ films in the a-MoS_2_/n-Si and a-MoS_2_/p-Si junctions, respectively, making them good candidates to explore the true origin of LPE properties in Si-based PSDs.

**Fig. 5:**
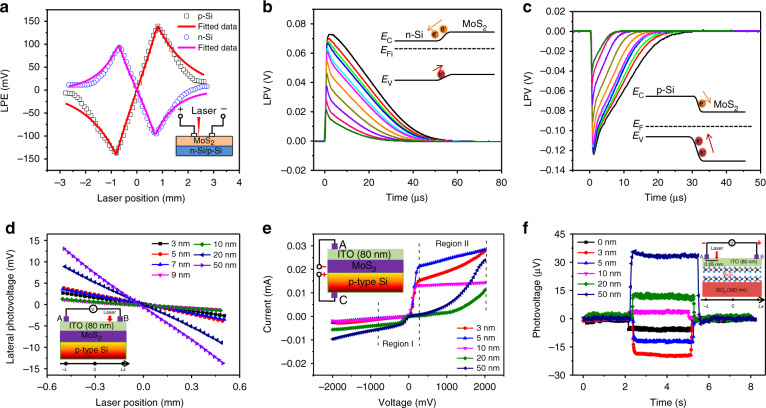
Large positional sensitivity with fast response time of MoS_2_-based PSD. **a** Dependence of the LPE on the illumination position relative to the location of electrodes in a-MoS_2_/n-Si (blue) and a-MoS_2_/p-Si (black) junctions. Variation in the LPE with time in **b** a-MoS_2_/n-Si and **c** a-MoS_2_/p-Si junctions. The insets show schematic diagrams of the energy band structure. **d** Dependence of LPE on the laser position for ITO/MoS_2_/p-Si heterojunctions with different MoS_2_ thicknesses. **e** Longitudinal I–V curves of ITO/MoS_2_ (3, 5, 10, 20, and 50 nm)/p-Si heterojunctions. **f** Time-dependent photovoltages in ITO/MoS_2_ (3, 5, 10, 20, and 50 nm)/SiO_2_ heterojunctions with illumination of a 532 nm laser^5,26^

Figure 5a shows that the a-MoS_2_/n-Si and a-MoS_2_/p-Si junctions have opposite LPEs, and the LPEs between the electrodes of the two junctions have a good linear dependence on the position of the laser irradiation point. The largest positional sensitivity observed in the a-MoS_2_/n-Si and a-MoS_2_/p-Si junctions is 183 and 145 mV mm^−1^, respectively.

When the a-MoS_2_ film is deposited on Si substrates, different band structures of a-MoS_2_/n-Si and a-MoS_2_/p-Si junctions are formed due to different Fermi levels of n-Si and p-Si, resulting in different orientations of the built-in field, as shown in the inset of Fig. 5b, c, respectively. The excited electrons and holes flow to the film side and then spread laterally in a-MoS_2_/p-Si and a-MoS_2_/n-Si junctions, respectively, providing different effects on the response time. Figure 5b, c show a variation in the response time in the a-MoS_2_ junctions. Due to the built-in field, minority carriers in the semiconductor will gather at the interface of the heterojunction to form an inversion layer. The thickness of the inversion layer at the SiO_2_/Si interface is less than 5 nm, and the room temperature mobilities of electrons and holes in the inversion layer are approximately 700 and 400 cm^2^ V^−1^ s^−1^, respectively. The mobility of electrons in a-MoS_2_ films deposited on p-Si substrates is approximately 415 cm^2^ V^−1^ s^−1^, and the mobility of holes in a-MoS_2_ films deposited on n-Si substrates is approximately 154 cm^2^ V^−1^ s^−1^. These results indicate that most of the photogenerated carriers diffuse laterally in the inversion layer with higher mobilities than in the a-MoS_2_ film. When the a-MoS_2_/n-Si junction is connected with a 300 Ω resistor, the rise time is ∼200 ns, and the relaxation time is reduced to approximately 5.8 μs. In the a-MoS_2_/p-Si junction, the fastest rise time is ∼150 ns, and the relaxation time is ∼2.1 μs, nearly three times faster than that in the a-MoS_2_/n-Si junction, which is consistent with the different mobilities of electrons and holes in the inversion layer at the interface between MoS_2_ and Si. These results clearly indicate that the interface of the a-MoS_2_/Si junction is responsible for the LPE.

Qiao et al. found that MoS_2_ undergoes n-type to p-type transformation with increasing thickness in the ITO/MoS_2_/p-Si heterojunction, and the LPE significantly improved in a wide spectrum response ranging from visible to near-infrared^5^. Figure 5d shows the LPE of ITO/MoS_2_/p-Si heterostructures under illumination with a 532-nm laser. When the thickness of MoS_2_ is less than 9 nm, the LPE first increases and then decreases with an increase in the film thickness. As the film thickness continues to increase, the LPE increases again, indicating that the thickness of MoS_2_ plays an important role in the transport of photogenerated carriers. To further elucidate the mechanism, the longitudinal *I*–*V* curves of the heterojunction were measured, as shown in Fig. 5e. With an increase in the thickness of MoS_2_, the Schottky barrier in region I (representing the characteristic between p-Si and MoS_2_) is enhanced, but in region II (representing the characteristic between the ITO layer and MoS_2_), it is reduced. When the thickness of MoS_2_ increases from 5 to 10 nm, the Schottky barrier in both regions I and II is reduced, and the carriers could hardly be transmitted from the MoS_2_ layer to the ITO layer, so the current is greatly decreased. As the thickness of MoS_2_ increases again, the Schottky barrier in region I still decreases. However, the Schottky barrier in region II is significantly improved, and the built-in field can accelerate photogenerated electron transfer from p-Si to the ITO layer, causing the LPE to rapidly increase again.

The photovoltaic response of ITO/MoS_2_/SiO_2_ samples with different MoS_2_ thicknesses was measured, as shown in Fig. 5f. Two electrodes with a pitch of 1.0 mm were plated on the surface of each sample, and a laser (532 nm, 10 mW) was used to irradiate at a point with a distance of 0.25 mm from the electrode. It was observed that when the thickness of MoS_2_ is less than 10 nm, the photovoltage is always negative, while the photovoltage of samples with thicker films changed from negative to positive and increased rapidly with increasing MoS_2_ thickness. These results indicate that the built-in field at the interface is reversed due to the n-type to p-type transformation of MoS_2_.

The built-in field at the interface of 2D materials and semiconductor substrates plays an important role in the performance of PSDs. The transmission direction of photogenerated carriers and response time can be effectively tuned by changing the band structure or thickness of the film, which is meaningful for the design of new PSDs.

## Conclusions and outlook

The rise of 2D materials has aroused great interest in new PSDs. To achieve higher-performance PSDs, various strategies have been used with different 2D materials. By studying the low-energy consumption, high sensitivity, and ultrafast response sensing systems based on 2D materials such as graphene and transition sulfides, the physical mechanisms of the large LPE and ultrafast response time in the PSD were elucidated. We believe that the high mobility and absorption of 2D materials are important factors to achieve a high-performance PSD. The high mobility provides a guarantee for the high-speed transmission of a large number of photogenerated carriers, which is important for the response speed of the PSD. High absorption can effectively increase the number of photogenerated carriers and enhance the sensitivity of the PSD. In addition, a PSD that combines 2D materials with various semiconductors not only effectively utilizes the high carrier mobility and light absorption of 2D materials but also uses the built-in field to spontaneously separate photogenerated carriers. We believe that the effective use of the characteristics of 2D materials to prepare high-sensitivity, fast-response, and other high-performance PSDs is the main research direction in the future. Combining the advantages of different 2D materials in one detector, such as using the high mobility of graphene and the suitable band structure of MoS_2_ to produce high-performance and low-consumption detectors, is also of great significance. Furthermore, there are still many challenges and opportunities for PSDs based on 2D materials in future research. For example, reducing the technical difficulty of large-area film preparation is a prerequisite for the development of multipoint and image detection functions. Because of the limitations of the energy band of Si and 2D materials, current PSDs based on 2D materials mainly absorb visible and near-infrared light, and the development of UV PSDs can provide a wider application range.
